# Derivation of Neural Progenitors and Retinal Pigment Epithelium from Common Marmoset and Human Pluripotent Stem Cells

**DOI:** 10.1155/2012/417865

**Published:** 2012-03-20

**Authors:** Laughing Bear Torrez, Yukie Perez, Jing Yang, Nicole Isolde zur Nieden, Henry Klassen, Chee Gee Liew

**Affiliations:** ^1^Stem Cell Center, Department of Cell Biology and Neuroscience, University of California, Riverside, Riverside, CA 92521, USA; ^2^Gavin Herbert Eye Institute, Department of Ophthalmology, School of Medicine, University of California, Irvine, Irvine, CA 92697, USA; ^3^Deptartment of Cell Therapy, Applied Stem Cell Technology Unit, Fraunhofer Institute for Cell Therapy and Immunology, Perlickstra*β*e 1, 04103 Leipzig, Germany

## Abstract

Embryonic and induced pluripotent stem cells (IPSCs) derived from mammalian species are valuable tools for modeling human disease, including retinal degenerative eye diseases that result in visual loss. Restoration of vision has focused on transplantation of neural progenitor cells (NPCs) and retinal pigmented epithelium (RPE) to the retina. Here we used transgenic common marmoset (*Callithrix jacchus*) and human pluripotent stem cells carrying the enhanced green fluorescent protein (eGFP) reporter as a model system for retinal differentiation. Using suspension and subsequent adherent differentiation cultures, we observed spontaneous *in vitro* differentiation that included NPCs and cells with pigment granules characteristic of differentiated RPE. Retinal cells derived from human and common marmoset pluripotent stem cells provide potentially unlimited cell sources for testing safety and immune compatibility following autologous or allogeneic transplantation using nonhuman primates in early translational applications.

## 1. Introduction

Novel applications of stem-cell-based therapies have revolutionized how degenerative diseases are approached. Given the propensity of stem cells to differentiate to neuronal pathways, diseases affecting the nervous system and associated tissues, such as the retina, are of great value. Retinal diseases, such as age-related macular degeneration (AMD), retinitis pigmentosa, and Stargardt disease, that render individuals functionally blind are commonly the result of impaired or complete loss of function of the photoreceptor cells or supporting retinal pigmented epithelium (RPE) [1–3]. To support *in vivo *transplantation, a readily available and efficient protocol for obtaining donor neural retinal and RPE cells is required.

Previous studies have demonstrated the capacity of human embryonic stem cells (HESCs) and human-induced pluripotent stem cells (HIPSCs) to differentiate into cells with RPE morphology, function, and molecular phenotypes [4, 5]. Thus far, HESC-, HIPSC- and fetal-derived RPE have been used to study the extent to which transplantation can correct retinal degenerative diseases [2, 5]. Preclinical studies in dystrophic rats have reported the ability of HESC-derived RPE cells to rescue visual function [1].

Before HESC or HIPSC derivatives can be used in clinical settings, safety and reproducibility of these cells must be vigorously tested in animal models. Although the use of transgenic mice has been of great value in early studies, cross-species differences often hamper efficacy and risk assessment in preclinical studies and are generally inadequate for evaluation of immunological responses. On the other hand, nonhuman primates provide valuable, and infrequently exploited, tools for extension of rodent results in models potentially more relevant to regenerative medicine. Due to their homology and highly similar physiology with humans, several species of monkeys have been used as preclinical nonhuman primate models. Recently, the common marmoset monkey (*Callithrix jacchus)* has been identified to be a cost-efficient and easily maintained nonhuman primate model of interest in biomedical research [6].

Derivation of Callithrix embryonic stem cells (CESCs) has opened up opportunities to study various aspects of early embryonic development pertinent to humans, as well as use of these cells to derive functional cell types for *in vitro* and *in vivo* studies [7, 8]. However there is a passage limit on long-term cultivation of CESC lines that have been created. It is therefore essential to utilize the lines that have been successfully derived in order to characterize their lineage-specific differentiation and explore their full potential.

Transgenic pluripotent stem cell lines carrying a marker gene are valuable for the study of differentiation potential and migration in host tissue. To test the function of transgenes in genetically modified ESCs, it is important to achieve stable gene expression during different stages of cell differentiation [9]. Here, we demonstrate the derivation of retina, including neural progenitor cells (NPCs) and retinal pigmented epithelium (RPE), from stable transfectants of both human and marmoset pluripotent stem cells carrying the enhanced green fluorescent protein (eGFP) reporter.

## 2. Materials and Methods

### 2.1. Derivation of Human Induced Pluripotent Stem Cells (HIPSCs)

Foreskin fibroblast cells (ATCC) were propagated in Dulbecco's Modified Eagle Medium (DMEM) supplemented with 10% fetal bovine serum (FBS), 1 mM Glutamax-I, and 1 mM nonessential amino acid (NEAA). 293FT cells were used as a packaging, cell line for generating retroviruses. 293FT were transfected with FuGENE HD with pMXS-OCT4, -SOX2 or -KLF4 plasmid, pHIT60 packaging and pVSV-G envelope construct. Medium-containing retroviruses were collected two days after-transfection. Foreskin fibroblast cells were infected with retroviruses and maintained in a 5% O_2_ incubator. Two days later, cells were replated on feeder layers and medium was changed to HIPSC medium (KnockOut DMEM/F12 supplemented with KnockOut Serum Replacement, 1 mM Glutamax-I, 1 mM NEAA, 55 mM 2-mercaptoethanol and 10 ng/mL FGF2). HIPSC colonies were picked using 200 *μ*L pipette tips four weeks after-transduction and maintained on matrigel as feeder-free cultures in StemPro (Invitrogen) or mTESR medium (Stem Cell Technologies). For subcultivation, HIPSCs were treated with accutase (Invitrogen) for 1 min, harvested by centrifugation, and replated onto new matrigel-coated dishes in StemPro medium. All cell lines were maintained in 37°C and 5% CO_2_.

### 2.2. Culture of Human Embryonic and Induced Pluripotent Stem Cells

Riv9 HIPSCs [10] were cultured in mTESR media (Stem Cell Technologies) on Geltrex-coated tissue-culture-treated dishes in 5% CO_2_ and 37°C. Cells were subcultivated every 5–7 days upon reaching 80–90% confluency by gently dislodging colonies using accutase and 15 mm glass beads. mTESR media were replenished daily.

### 2.3. Culture of Callithrix Embryonic Stem Cells (CESCs)

Cjes001 Callithrix embryonic stem cells (CESCs) [8] were maintained on irradiated mouse embryonic fibroblast (MEF) feeders in growth medium: knockout high glucose DMEM supplemented with 15% KOSR, 1% nonessential amino acids, 2 mM of Glutamax-I, 0.1 mM b-mercaptoethanol, and 10 ng/mL bFGF. The cells were routinely passaged with 0.25% trypsin/EDTA at a ratio of 1 : 5–1 : 8 every 5–7 days.

### 2.4. Differentiation of HESCs, HIPSCs, and CESCs

HESC and HIPSC cultures previously maintained in mTeSR were treated with rock inhibitor (RI) for 1 hour prior to dissociation into single cells with 0.25% trypsin/EDTA. Cells were resuspended in STEMPRO media lacking bFGF and replated onto non-tissue-culture-treated Petri dishes. Cjes001 cells were trypsinized, pelleted, and differentiated in CESC media lacking bFGF on non-tissue-culture-treated Petri dishes. The differentiating cells formed aggregates termed embryoid bodies (EBs), consisting of cells representative of three differentiated germ layers.

### 2.5. Nucleofection of HESC and HIPSCs

Trypsinized single-cell suspension was resuspended in 100 *μ*L of prewarmed human stem cell nucleofector solution 1 (Lonza). The cells in human stem cell Nucleofector solution 1 were then transferred to a cuvette and 4 *μ*g of plasmid DNA was added. The cuvette was gently swirled, tapped twice on the bench, inserted into the cuvette holder of the Lonza Amaxa Nucleofector II Device, and nucleofected using B-16 program. The nucleofected cells were recovered in prewarmed media and incubated at 37°C for 10 minutes prior to replating on feeder cells.

### 2.6. Flow Cytometry Analysis

Nucleofected cells were dissociated with 0.25% trypsin/EDTA. Cell pellets were then resuspended in 250 *μ*L of wash buffer and subjected to flow cytometry on a SC Quanta flow cytometer (Beckman Coulter).

### 2.7. Reverse Transcription-Polymerase Chain Reaction (RT-PCR)

Total RNA was isolated using the ZR RNA MicroPrep kit (Zymo Research). RNA concentration was measured using a NanoDrop spectrophotometer (Thermo Scientific). First-strand cDNA synthesis was performed using iScript cDNA Synthesis kit (Bio-Rad). Following cDNA synthesis semiquantitative RT-PCR was performed. Each RT-PCR reaction consisted of PCR master mix, 0.3 pM forward primer, and 0.3 pM reverse primer, and cDNA, RT-PCR amplifications were initiated at 95°C for 5 mins followed annealing and extension. The forward/reverse primers and annealing temp used for primate genes were ACTB 5′-ATCTGGCACCACACCTTCTACAATGAGCTGCG-3′ and 3′-CGTCATACTCCTGCTTGCTGATCCACATCTGC-5′, 58°C; LRAT 5′-CTCATCCTGGGCGTTATTGT-3′ and 3′-CCAGCCAT CCAT AGGA AGAA-5′, 49°C; NEUROD1 5′-AAGCCATGAACGCAGAGGAGGACT-3′ and 3′-AGCTGTCCATGGTACCGTAA-5′, 55°C. Following RT-PCR amplification products were observed by gel electrophoresis on a flash gel (Lonza).

### 2.8. Real-Time Quantitative Polymerase Chain Reaction (Q-PCR) Analysis

Q-PCR was performed using the Assay-on-Demand technology (Applied Biosystems). Each reaction consisted of 2.5 *μ*L of 2x TaqMan master mix, 0.25 *μ*L of TaqMan probes, 1.25 *μ*L of water, and 1 *μ*L of cDNAs (100 ng, standardized based on housekeeping gene controls). PCR amplifications were initiated at 95°C for 10 mins followed by 42 cycles of 95°C for 15 seconds and 60°C for 60 seconds. PCR reactions for each sample were performed using 384-well real-time CFX384 thermocycler (BioRad). The Q-PCR data were analyzed using the comparative C_T_ method. Q-PCR was performed in duplicates from three different cDNA samples.

### 2.9. Immunocytochemistry

Cells were washed twice with PBS w/o Mg^2+^ and Ca^2+^ and fixed with 4% paraformaldehyde for 10 minutes. The fixed cells were then washed again with PBS w/o Mg^2+^ and Ca^2+^. Next, fixed cells were blocked for 30 minutes with PBS w/o Mg^2+^- and Ca^2+^-based wash buffer containing 1% donkey serum and 0.1% Triton-X. Fixed cells were then incubated overnight at 4°C in primary antibodies: Vimentin, MAP2 (Cell Signaling), OCT4, GFAP (Santa Cruz), SOX2 (R&D Systems), SSEA3 (Millipore), and TUJ1 (Covance). After overnight incubation at 4°C, primary antibody solution was removed. Cells were subsequently washed twice with wash buffer. Secondary antibodies were then added to the stained cells in wash buffer and incubated in the dark at 25°C for 1 hour. Following secondary antibody incubation the cells were washed twice with wash buffer with a 5 min incubation step during each wash. Cells were mounted in DAPI mounting solution (Vectashield) and imaged using the Nikon Ti Eclipse and NIS-elements imaging software.

## 3. Results

### 3.1. Derivation of eGFP-Expressing Callithrix and Human Pluripotent Stem Cell Lines

**Figure 1:**
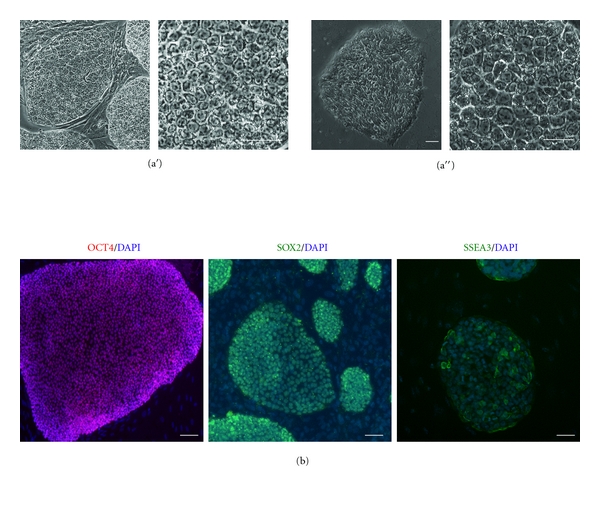
Cjes001 common marmoset embryonic stem cells (CESCs) closely resemble the morphology of human pluripotent stem cells. (a′) Colonies of CESCs grown on irradiated feeder cells (4x magnification, left) and morphology of individual CESCs at 20x magnification (right). (a′′) Morphology of Riv9 HIPSC colony (4x mag, left) and individual cells within the colony (20x, right). (b) Immunocytochemical analysis of CESCs showing nuclear localization of OCT4 (red), SOX2 (green), and stage-specific embryonic antigen-3 (SSEA3, green). Cell nuclei were counterstained with DAPI. Scale bars, 50 *μ*m.

**Figure 2:**
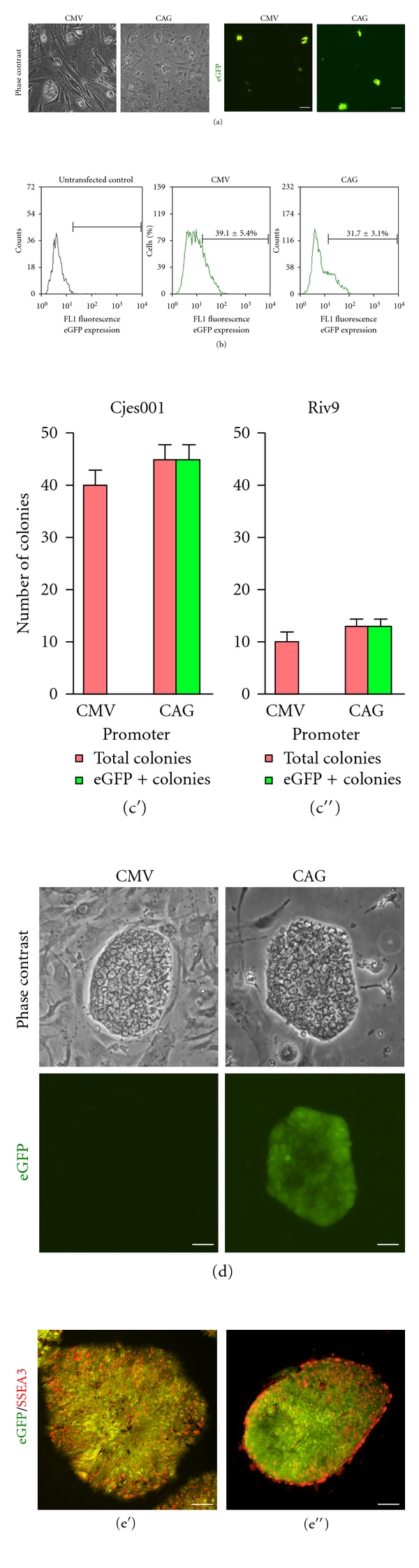
Transfection of cjes001 common marmoset CESCs and Riv9 HIPSCs. Micrographs (a) and FACS histograms (b) enumerating the percentage of eGFP-positive (eGFP +ve) cjes001 CESCs 24 hours after-transfection. (c) Numbers of drug-resistant and eGFP-expressing colonies formed after two weeks were scored for the stable transfection assay. (d) eGFP expression was lost in all pCMV-transfected clones. In contrast, all puromycin-resistant colonies were also eGFP +ve. Cjes001 (e′) and Riv9 clone (e′′) retained ubiquitous and constitutive eGFP expression while continuously express undifferentiated stem cell marker SSEA3 (red). Scale bars, 50 *μ*m.

Cjes001 Callithrix embryonic stem cells (CESCs) displayed similar morphology to Riv9 human induced pluripotent stem cells (HIPSCs; Figure 1(a′)). The undifferentiated cjes001 also expressed OCT4 and SOX2 transcription factors and stage-specific embryonic antigen-3 (SSEA3; Figure 1(b)). In these characteristics, marmoset ESCs closely resemble HIPSCs and HESCs [11]. In a pilot study, we compared the efficacy of the CMV and CAG promoters in deriving stable transfectants in cjes001 CESCs and Riv9 HIPSCs. These two promoters were previously described as strong promoters in human embryonic stem cells (HESCs) and HIPSCs [10, 12], but their activities in CESCs were not known. Single-cell suspensions were nucleofected, replated on feeders, and examined for transient transfection efficiency the next day (Figure 2(a)). Flow cytometry analysis demonstrated that 39.1 ± 5.4% and 31.7 ± 3.1% cjes100 cells transfected with pCMV-eGFP and pCAG-eGFP expressed eGFP marker gene, respectively (Figure 2(b)). Thus our data suggests that marmoset ESCs yielded higher transient transfection efficiency compared to HIPSCs [10].

Stable transfectants that survived in the presence of antibiotic selection appeared within two weeks after-nucleofection. The frequency with which stably transfected clones could be recovered during the drug selection process varied among HIPSCs and CESCs. Optimal doses for drug selection were constructed from kill curves with Geneticin (G418) and puromycin. 500 *μ*g/*μ*L of G418 and 1.5 *μ*g/*μ*L puromycin were sufficient to select for transfectants in cjes001, while 200 *μ*g/*μ*L of G418 and 1 *μ*g/*μ*L of puromycin specifically selected for stable integrants in Riv9 with minimal background of nonresistant cells. We observed the presence of distinct eGFP-expressing colonies in pCAG-transfected Riv9 and cjes001 (Figures 2(c) and 2(d)). In contrast, none of the cells were eGFP positive in clones carrying the CMV promoter, confirming previous reports that CMV promoter is highly silenced in pluripotent stem cells [12]. These transgenic eGFP-expressing pCAG-transfected clones continued to express SSEA3 a month after cultivation (Figure 2(e)). Thus we demonstrated that transgenic HIPSCs and CESCs maintained their pluripotent potential.

### 3.2. Differentiation of Retinal Cell Precursors

**Figure 3:**
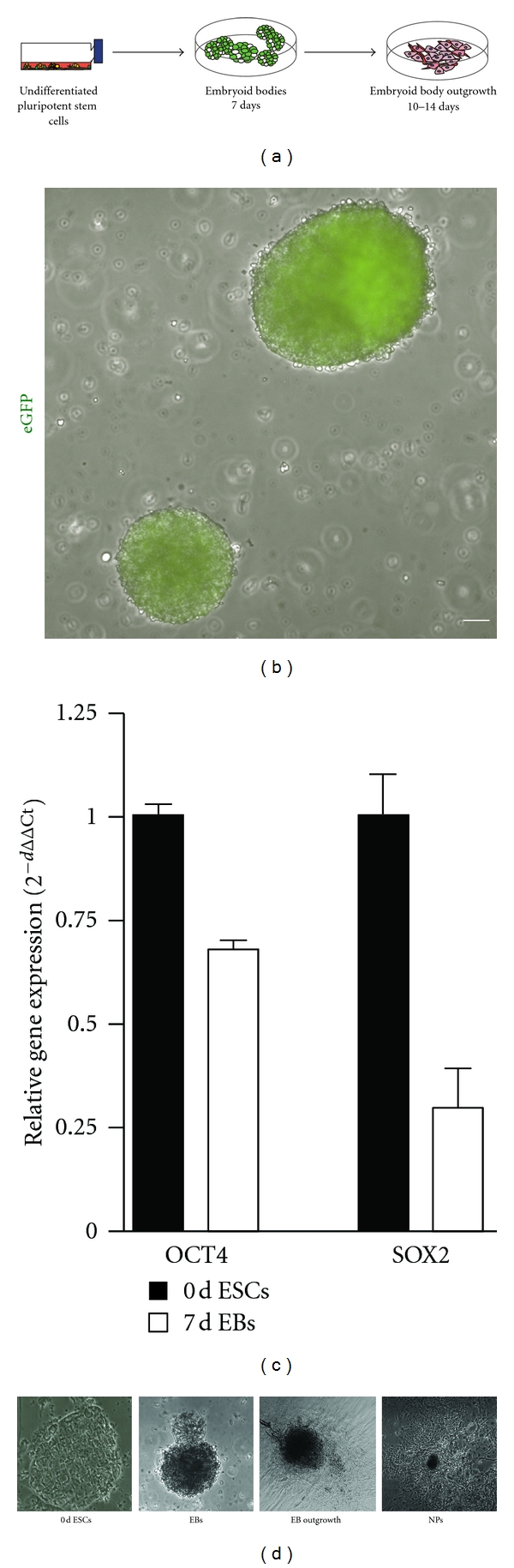
Differentiation of cell progenitors associated with the central nervous system (CNS) and the neural retina. (a) Experimental overview for *in vitro* differentiation of CESCs. (b) Constitutive eGFP expression in differentiated aggregates of cjes001 EBs. (c) Q-PCR analysis of OCT4 and SOX2 pluripotency markers in undifferentiated cjes001 (0-day ESCs) and 7-day EBs. (d) Changes in morphology during *in vitro* differentiation. Arrowheads indicate EB outgrowth observed 1 week after replating. Neurites resembling neural progenitors (NPs) were formed 10–14 days after replating. Scale bars, 50 *μ*m.

We next sought to characterize the potential of these eGFP-expressing transgenic human and nonhuman primate ESCs to differentiate into cells related to retinal lineage. Cjes001 and Riv9 cells were detached, transferred to non-tissue-culture-treated dishes, and differentiated in media lacking bFGF to facilitate spontaneous differentiation of cells (Figure 3(a)). Suspension cultures prompted the formation of free-floating aggregates termed embryoid bodies (EBs). eGFP expression was retained in these cells during *in vitro* differentiation, indicating stable transgene integration (Figure 3(b)). Q-PCR analysis revealed downregulation of pluripotency markers OCT4 and SOX2 in EBs (Figure 3(c)).

**Figure 4:**
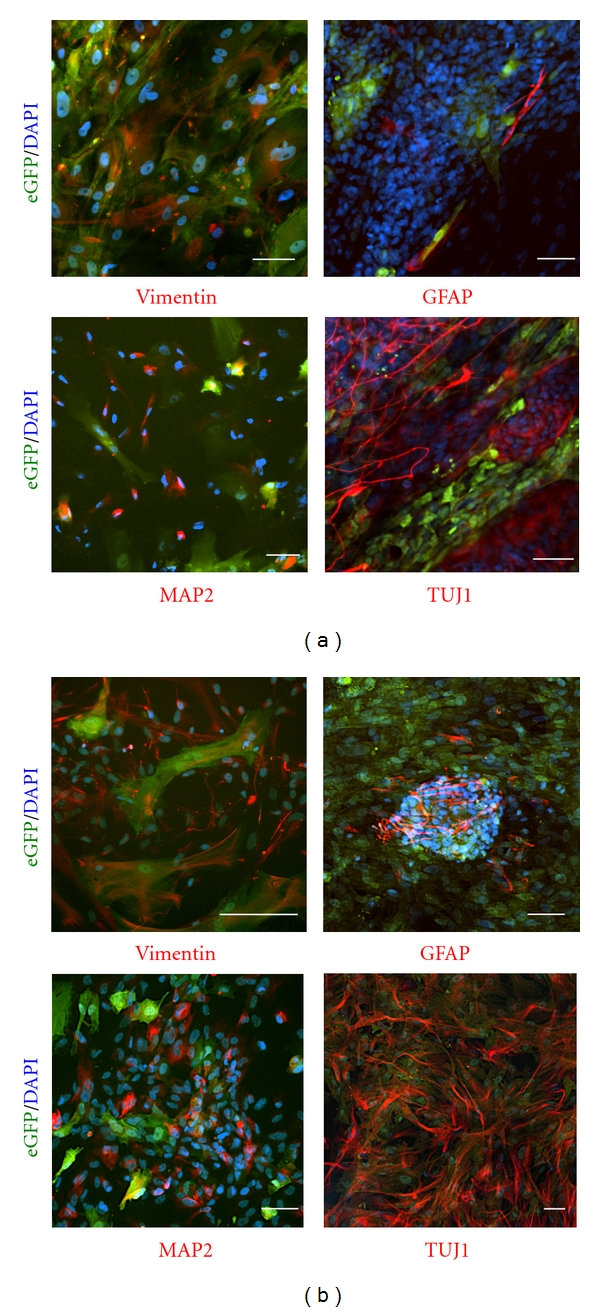
Expression of neural lineage-related cytoskeletal proteins in cjes001 CESCs (a), Riv9 HIPSCs (b). Immunocytochemistry using antibodies specific for neural markers are shown in red. Green fluorescence indicates eGFP expression in pCAG-transfected differentiated derivatives. Scale bars, 50 *μ*m.

To investigate the effect of transgene expression on central nervous system (CNS) and retinal differentiation, we replated EBs on matrigel for further differentiation in monolayer cultures. Cells spread out, expanded to monolayer as EB outgrowth, and readily underwent further differentiation (Figure 3(d)). Stably transfected eGFP-expressing cjes001 CESCs differentiated to neural progenitor cells (NPCs) following *in vitro* differentiation. Notably, cellular morphologies of cells were similar to those observed in primary or HESC-derived neural progenitor cultures [13, 14]. Immunocytochemistry analysis revealed the expression of markers representative of different stages of neural lineage commitment in EB outgrowth, including the immature neural cell marker Vimentin (Figure 4(a)). Cells from EB outgrowth also showed immunoreactivity for gial fibrillary acidic protein (GFAP), an intermediate filament specific for astrocytes in CNS and Muller cells in retina. Cells immunoreactive for cytoplasmic microtubule-associated protein 2 (MAP2) and *β* III-tubulin (TUJ1), two markers of committed neural cells, were first observed two weeks after replating.

We compared the propensity of neural and retinal lineage differentiation in marmoset cjes001 CESCs to Riv9 HIPSCs. Reminiscent of spontaneous differentiation in marmoset cells, human pluripotent stem cells gave rise to cells with neuron-like morphologies. Nevertheless, we observed an increase in Vimentin, MAP2, TUJ1, and GFAP protein expression in Riv9 EB outgrowth (Figure 4(b)). Neural clusters possessed long processes and intense filamentous staining for TUJ1, indistinguishable from those of HESCs [15, 16]. In addition, as they emerged, GFAP-expressing cells were self-organized into filamentous aggregates, suggesting a more mature differentiation stage of HIPSC-derived neural cells. Taken together, these results indicate that HESCs and HIPSCs were predisposed to differentiate towards a neural lineage compared to marmoset ESCs.

### 3.3. Isolation of Retinal Pigment Epithelium

**Figure 5:**
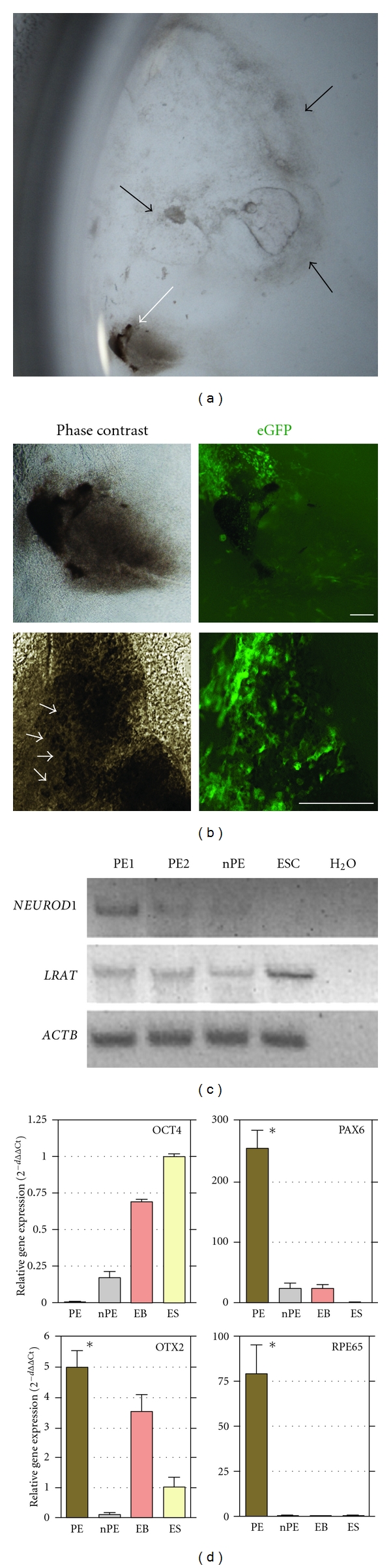
Differentiation of retinal pigmented epithelium (RPE) from Callithrix ESCs. (a) Stereoscopic image of cell outgrowth following EB replating. The white arrows indicate the visible pigmented area derived from an area of EB outgrowth. Black arrows indicate the colonies that did not develop to RPE structures. (b) Phase contrast and green fluorescence of the pigmented epithelium in RPE patch-like structures. The white arrowheads indicate the presence of putative RPE cells with typical pigmented cobblestone-like morphology. Scale bars, 200 *μ*m. (c) Semiquantitative PCR analysis of manually picked clusters of pigmented epithelium (PE1 and PE2), nonpigmented cells (nPE), and undifferentiated ESCs. Water (H_2_O) only was included as negative control. (d) Relative expression levels of OCT4, PAX6, OTX2s and RPE65 mRNA in PE, nPE, embryoid bodies (EB), and undifferentiated ES cells (ES). Mean normalized expression of each target gene is relative to ACTB and GAPDH housekeeping genes. Error bars represent standard deviation. Asterisk shows significant difference of PAX6, OTX2, and RPE65 expression in PE clusters, *P* < 0.05.

We consistently observed the appearance of pigmented cell colonies during the cell outgrowth from the EB clusters in cjes001 and Riv9. This phenomenon was strikingly similar to previous observation of retinal pigmented epithelium (RPE) present in confluent cultures derived from various HESCs and HIPSC lines [17, 18]. In our case, the most densely pigmented cells were located in the periphery of the EB clusters (Figure 5(a)). On average, less than 20% of cjes001 EB colonies differentiated to RPE structures. These CESC-derived pigmented cells exhibited cobblestone morphology, confirming the existence of RPE progenitors (Figure 5(b)).

To further examine the identity of these pigmented cells, we hand-picked and isolated the pigmented epithelium (PE) foci using pipette tips and examined the gene expression patterns. Although LRAT mRNA was observed in undifferentiated ESC sample, similar to previous report in HESCs [18], its expression was enriched in manually picked PE in comparison to nonpigmented area (nPE; Figure 5(c)). We also detected the expression of bHLH transcription factor NEUROD1, suggesting the presence of terminally differentiated neurons and thus the formation of an retina niche in the isolated PE cell layers. As revealed by quantitative PCR analysis, isolated RPE acquired expression for transcription factors associated with general neural retina induction (PAX6), eye field specification (OTX2), and retinal pigment epithelium (RPE65; Figure 5(d)). Notably, there was a complete loss of OCT4 mRNA expression in RPE, indicating the absence of residual undifferentiated stem cells.

## 4. Discussion

A key challenge in early translational research using human stem cells is the availability of a reliable host model to evaluate long-term benefits in clinical applications. Nonhuman primates are good candidates for testing the safety and feasibility of experimental protocols prior to cell replacement therapies in humans. Previous reports, as well as more recent studies, are beginning to reveal that stem cells can ameliorate the consequences of various degenerative diseases in nonhuman primates [19]. While the evidence for human pluripotent stem cell-derived retinal neural and RPE cells is burgeoning, the equivalent differentiation propensity in marmoset ESCs has not been previously explored.

The ability to genetically manipulate nonhuman primate embryonic stem cells is central in our efforts to harness their enormous potential for use in regenerative medicine.   Whereas transgenic marmoset offspring have been generated using self-inactivating lentivirus [6], to our knowledge this study is the first to report derivation of transgenic Callithrix embryonic stem cell lines. Although lentiviral infection has proven efficient in generating stable integrants, its application can be hampered by several challenges such as size limitations on inserted DNA and the time-consuming production of vectors. Here we report that the use of a plasmid harboring the CAG promoter resulted in ubiquitous and highly stable eGFP expression in marmoset and human pluripotent stem cells. Our finding also underscores the importance of the choice of promoter in engineering stable cell lines, as the activity of the CMV promoter was completely silenced after several cell divisions.

The present study demonstrates the derivation of retinal neural cells and pigmented epithelium from stable eGFP-expressing transfectants. Our success with deriving transgenic Callithrix embryonic stem cell clones allows the use of these reporter cell lines to follow and track transplanted cells in preclinical studies. Furthermore, targeted gene knockdown could be developed using overexpression or short hairpin RNA interference (shRNAi) vectors [20] to study human diseases involving the loss of function of specific genes in nonhuman primates, including Huntington's disease [21], spinal cord injury [22], and Parkinson's disease [23].

Commitment toward retinal lineage occurs as a stepwise process. RPE, despite its nonneural phenotype, is anatomically and developmentally close to the neural retina [24]. Our results suggest that different types of neural cells in the retina, as well as RPE structures *in vitro*, result from a normal developmental pathway which can be replicated using marmoset and human pluripotent stem cells in suspension cultures. Consistent with Osakada's finding [25], we did not detect any RPE-like pigmented foci in cells directly differentiated from monolayer cultures. Our finding is a necessary prerequisite for therapeutic strategies based on cell enrichment from human and nonhuman primate ESCs as a source of donor retinal cell types.

In order to achieve the long-term goal of utilizing pluripotent stem cells from nonhuman primates, methods for optimizing NPCs and RPE formation from CESCs are required. We found that Riv9 HIPSCs showed a higher incidence of differentiation towards neural lineage, further supporting the notion that human pluripotent stem cells assume a default neural default pathway in the absence of extrinsic factors during *in vitro* differentiation [26]. Another possible explanation of lower neural commitment of cjes001 CESC line includes their readily enhanced differentiation potential into germ cells as previously reported [7]. Hence, early neutralization may increase the yield of neural precursors from cjes001 CESCs.

As ES cell lines are derived from a genetically heterogeneous population, there may be biological variations, heterogeneity, genetic, and epigenetic differences between different ESC lines. Our findings thus underscore the necessity of establishing and screening novel nonhuman primate stem cell lines for lineage-specific differentiation. Moreover, the availability of marmoset IPSCs [27] would accelerate the advance of preclinical studies in regenerative medicine, allowing the assessment of safety and efficacy of allogenic and xenogenic transplantation for various retinal degenerative diseases.
